# Models of Affective Decision Making: How Do Feelings Predict Choice?

**DOI:** 10.1177/0956797616634654

**Published:** 2016-04-12

**Authors:** Caroline J. Charpentier, Jan-Emmanuel De Neve, Xinyi Li, Jonathan P. Roiser, Tali Sharot

**Affiliations:** 1 Institute of Cognitive Neuroscience, University College London; 2 Affective Brain Lab, Department of Experimental Psychology, University College London; 3 Saïd Business School, University of Oxford

**Keywords:** decision making, feelings, subjective well-being, value, utility, prospect theory

## Abstract

Intuitively, how you feel about potential outcomes will determine your decisions. Indeed, an implicit assumption in one of the most influential theories in psychology, prospect theory, is that feelings govern choice. Surprisingly, however, very little is known about the rules by which feelings are transformed into decisions. Here, we specified a computational model that used feelings to predict choices. We found that this model predicted choice better than existing value-based models, showing a unique contribution of feelings to decisions, over and above value. Similar to the value function in prospect theory, our feeling function showed diminished sensitivity to outcomes as value increased. However, loss aversion in choice was explained by an asymmetry in how feelings about losses and gains were weighted when making a decision, not by an asymmetry in the feelings themselves. The results provide new insights into how feelings are utilized to reach a decision.

How would you feel if you won an award for outstanding professional achievement? How would you feel if your marriage broke apart? Intuitively, answers to these questions are important, as they should predict your actions. If the prospect of losing your spouse does not fill you with negative feelings, you may not attempt to keep your marriage intact. But how exactly do feelings associated with possible outcomes relate to actual choices? What are the computational rules by which feelings are transformed into decisions? While an expanding body of literature has been dedicated to answering the reverse question, namely how decision outcomes affect feelings (Carter & McBride, 2013; Kassam, Morewedge, Gilbert, & Wilson, 2011; Kermer, Driver-Linn, Wilson, & Gilbert, 2006; McGraw, Larsen, Kahneman, & Schkade, 2010; Mellers, Schwartz, Ho, & Ritov, 1997; Rutledge, Skandali, Dayan, & Dolan, 2014; Yechiam, Telpaz, & Hochman, 2014), little is known about how feelings drive decisions about potential outcomes.

In the present study, we examined whether feelings predict choice and built a computational model that specified this relationship. We turned to prospect theory (Kahneman & Tversky, 1979; Tversky & Kahneman, 1986, 1992) as a starting point in this research. Prospect theory was not derived by eliciting people’s feelings to predict choice, but rather by observing people’s choices in order to estimate the subjective value associated with possible outcomes. An implicit assumption of the theory, however, is that subjective value (utility) is a proxy for feelings, which in turn govern choice; “humans described by Prospect Theory are guided by the immediate emotional impact of gains and losses” (Kahneman, 2011, p. 287). This suggests that if one measures a person’s feelings associated with different outcomes, one should be able to generate that person’s utility function and use it to predict his or her choices. While prospect theory is one of the most influential theories in economics and psychology, this implicit assumption has never been empirically tested. Thus, it is not known if and how feelings guide choice.

To address this question, we conducted three studies, in which we asked participants to report how they felt or expected to feel after winning or losing different amounts of money (the main study is presented here and the two extension and replication studies are presented in the Supplemental Material available online). We used those self-reported feelings to create a *feeling function*, a function that best relates feelings (expected or experienced) to objective value. Next, we used this function to predict participants’ choices in a different decision-making task. Our findings were replicated in all three studies.

An intriguing question was what such a feeling function would look like. One possibility is that it would resemble the value function in prospect theory, which relates the subjective value estimated from choice data to objective value. First, for most people, the value function is steeper for losses than for gains. This results in loss aversion, such that the absolute subjective value of losing a dollar is greater than that of winning a dollar. Yet while it appears that “losses loom larger than gains” (Kahneman & Tversky, 1979, p. 279), it is not known whether the impact of a loss on one’s feelings is greater than the impact of an equivalent gain. Alternatively, it is possible that the impact of gains and losses on feelings is similar but that the weight given to those feelings differs when one makes a choice.

Second, prospect theory’s value function is convex in the loss domain while concave in the gain domain (such that it resembles an S). The curvature of the function in both domains represents the notion of diminishing sensitivity to changes in value as gains and losses increase. In other words, the subjective value of gaining (or losing) $10 is smaller than twice that of gaining (or losing) $5. This diminishing sensitivity results in risk aversion in the gain domain and risk seeking in the loss domain, with individuals tending to choose a small sure gain over a high but risky gain, but a high risky loss over a small sure loss. We examined whether our feeling function was also concave for gains and convex for losses, which would imply that similar to subjective value, feelings associated with gains and losses are less sensitive to outcome value as gains and losses increase. That is, the impact of winning (or losing) $10 on feelings is less than twice the impact of winning (or losing) $5.

Once feelings were modeled using this feeling function, we asked whether they could predict choice. Understanding how explicit feelings relate to behavior has important real-world implications for domains ranging from policy to industry.

## Method

### Participants

Fifty-nine healthy volunteers (24 males, 35 females; mean age = 23.94 years, age range 19–35) from the University College London Subject Pool were recruited to take part in the experiment. Sample size was determined using a power analysis (G*Power Version 3.1.9.2; Faul, Erdfelder, Lang, & Buchner, 2007) based on previous studies that have investigated the link between decision outcomes and self-reported feelings using within-subjects designs. Effect sizes (Cohen’s *d*) in those studies ranged from 0.245 to 0.798, with a mean of 0.401 (Harinck, Van Dijk, Van Beest, & Mersmann, 2007; Kermer et al., 2006; Yechiam et al., 2014). We determined that a sample size of 59 participants would achieve 85% power to detect an effect size of 0.401 with an alpha of .05.

Three participants were excluded: 1 whose feeling ratings showed no variation at all, 1 whose data from the gambling task were lost, and 1 who failed to complete more than 50% of the trials in the gambling task. Final analyses were therefore run on 56 participants (22 males, 34 females; mean age = 23.91 years, age range 19–35). All participants gave written informed consent and were paid for their participation. All started the experiment with an initial endowment of £12 and were paid according to their choices on two randomly chosen trials (across the two tasks) at the end of the experiment. The experiment was approved by the departmental ethics committee at University College London.

### Behavioral tasks

Participants completed two tasks, the feelings task and the gambling task, the order of which was counterbalanced.

#### Feelings task

**Fig. 1.**
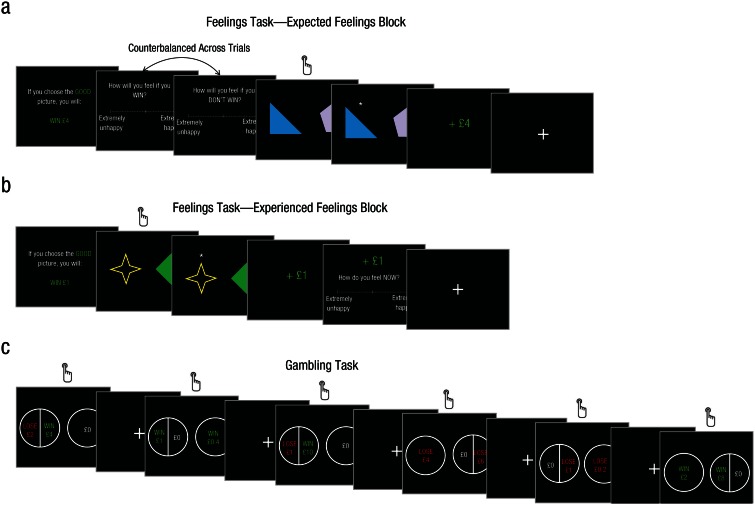
Example trial sequences from the (a, b) feelings task and (c) gambling task. Participants completed two versions of the feelings task in different blocks. On both (a) expected-feelings and (b) experienced-feelings blocks (one trial of each is shown here), participants won or lost money if they correctly chose between one of two shapes. The amount was specified at the start of the trial, along with whether the trial involved winning money (as shown here) or losing money. In all feelings trials, they rated how happy they would be if they won or lost, with the sole difference being that they rated their feelings before choosing between the stimuli on expected-feelings trials but after choosing between the stimuli on experienced-feelings trials. On each trial of (c) the gambling task (six trials of which are shown here), participants chose between a sure option (e.g., getting £0) and a risky option (e.g., winning £2 or losing £4). The same amounts were used in the gambling and feelings tasks.

In the feelings task, participants completed four blocks of 40 to 48 trials each, in which they reported either expected (Fig. 1a) or experienced (Fig. 1b) feelings associated with a range of wins and losses (between £0.2 and £12) or with no change in monetary amount (£0). At the beginning of each trial, participants were told how much was at stake and whether it was a win trial (e.g., “If you choose the GOOD picture, you will: WIN £10”) or a loss trial (e.g., “If you choose the BAD picture, you will: LOSE £10”). On each trial, their task was to make a simple arbitrary choice between two different geometrical shapes. Participants were told that one stimulus was randomly associated with a gain or loss (between £0.2 and £12) and the other stimulus with no gain and no loss (£0). Each stimulus was presented only once across the entire task so there was no way for participants to learn which stimulus was associated with a better outcome. The probability of sampling each amount was controlled to ensure that each gain and each loss from the range was sampled twice in each block: In one instance, the outcome was the amount at stake (win/loss), and in the other, the outcome was £0 (no win/no loss).

In two of the four blocks (counterbalanced order), participants reported their expected feelings prior to choosing between the two stimuli (Fig. 1a), and in the other two blocks, they reported their experienced feelings after choosing between the two stimuli (Fig. 1b). Participants reported their expected feelings by answering one of four questions asking how they would feel if they “win,” “lose,” “don’t win,” or “don’t lose” (the order of win/lose and don’t-win/don’t-lose questions was counterbalanced across trials). In experienced-feelings blocks, participants answered the question “How do you feel now?” All feelings were rated using a subjective rating scale ranging from *extremely unhappy* to *extremely happy*. Expected and experienced feelings were collected in different blocks to ensure participants did not simply remember and repeat the same rating. The choice between the two geometrical shapes was arbitrary and implemented simply in order to have participants actively involved with the outcomes.

#### Gambling task

Participants also completed a probabilistic-choice task (Fig. 1c) in which they made 288 to 322 choices between a risky 50-50 gamble and a sure option. Importantly, all the amounts used in the gambling task were the same as those used in the feelings task (between £0.2 and £12), so feelings associated with these outcomes could be combined to predict gambling choice. There were three gamble types: mixed (participants had to choose between a gamble with a 50% chance of a gain and 50% chance of a loss, or a sure option of £0), gain only (participants had to choose between a gamble with a 50% chance of a high gain and a 50% chance of £0, or a sure, smaller gain), and loss only (participants had to choose between a gamble with 50% chance of a high loss and 50% chance of £0, or a sure, smaller loss). According to prospect theory, these three types of choices are essential to estimate loss aversion, risk preference for gains, and risk preference for losses, respectively.

### Feeling-function models

The impact of outcome on feelings was calculated relative to three different baselines: difference from the midpoint of the rating scale, difference from the rating reported on the previous trial (for experienced feelings only), and difference from the corresponding zero outcome. These were calculated for each win and loss amount, for expected and experienced feelings separately. Using each of the three methods, we fit 20 feeling-function models (10 for expected feelings and 10 for experienced feelings) for each participant to explain how feelings best related to value outcomes:

Feeling Model1:F(x)=βx

$$\mathrm{Feeling Model}\;2:F\left(x\right)=\{\begin{array}{c} \beta_{\mathrm{gain}}x,x>0\\ \beta_{\mathrm{loss}}x,x<0 \end{array}$$

$$\mathrm{Feeling Model}\;3:F\left(x\right)=\{\begin{array}{c} \beta {\left(\left|x\right|\right)}^{\rho },x>0\\ -\beta {\left(\left|x\right|\right)}^{\rho },x<0 \end{array}$$

$$\mathrm{Feeling Model}\;4:F\left(x\right)=\{\begin{array}{c} \beta_{\mathrm{gain}}{\left(\left|x\right|\right)}^{\rho },x>0\\ -\beta_{\mathrm{loss}}{\left(\left|x\right|\right)}^{\rho },x<0 \end{array}$$

$$\mathrm{Feeling Model}\;5:F\left(x\right)=\{\begin{array}{c} \beta {\left(\left|x\right|\right)}^{\rho_{\mathrm{gain}}},x>0\\ -\beta {\left(\left|x\right|\right)}^{\rho_{\mathrm{loss}}},x<0 \end{array}$$

$$\mathrm{Feeling Model}\;6:F\left(x\right)=\{\begin{array}{c} \beta_{\mathrm{gain}}{\left(\left|x\right|\right)}^{\rho_{\mathrm{gain}}},x>0\\ -\beta_{\mathrm{loss}}{\left(\left|x\right|\right)}^{\rho_{\mathrm{loss}}},x<0 \end{array}$$

$$\mathrm{Feeling Model}\;7:F\left(x\right)=\{\begin{array}{c} \beta x+\varepsilon ,x>0\\ \beta x-\varepsilon ,x<0 \end{array}$$

$$\mathrm{Feeling Model}\;8:F\left(x\right)=\{\begin{array}{c} \beta_{\mathrm{gain}}x+\varepsilon ,x>0\\ \beta_{\mathrm{loss}}x-\varepsilon ,x<0 \end{array}$$

$$\mathrm{Feeling Model}\;9:F\left(x\right)=\{\begin{array}{c} \beta x+\varepsilon_{\mathrm{gain}},x>0\\ \beta x-\varepsilon_{\mathrm{loss}},x<0 \end{array}$$

$$\mathrm{Feeling Model}\;10:F\left(x\right)=\{\begin{array}{c} \beta_{\mathrm{gain}}x+\varepsilon_{\mathrm{gain}},x>0\\ \beta_{\mathrm{loss}}x-\varepsilon_{\mathrm{loss}},x<0 \end{array}$$

In all these models, x represents the value (from −12 to −0.2 for losses and from 0.2 to 12 for gains) and F the associated feeling. The slope between feelings and values is represented by the parameter β estimated as a single parameter in all odd-numbered models, or separately for losses and gains in all even-numbered models. If loss aversion is reflected in feelings, β_loss_ should be significantly greater than β_gain_, and even-numbered models should perform better overall. Similar to the curvature parameter of the value function in prospect theory, ρ reflects the curvature of the feeling function, that is, the fact that feelings become more or less sensitive to changes in value as absolute value increases (Feeling Models 3 to 6). In Feeling Models 5 and 6, the curvature is estimated separately in the gain and loss domains. If the feeling function is S-shaped (concave function for gains and convex function for losses), ρ values should be significantly smaller than 1. To ensure that a function with curvature fit the feelings data better than a simple linear function with an intercept, we defined Feeling Models 7 to 10 (as respective comparisons for Feeling Models 3–6); in these models, ε represents the intercept, or the offset (positive for gains, negative for losses) where feelings start for values close to £0. All these models were estimated in MATLAB (The MathWorks, Natick, MA) using a maximum-likelihood estimation procedure (Myung, 2003). Bayesian information criterions (BICs) were calculated for each participant and model, and then summed across participants (see the Supplemental Material for details). Lower BICs indicate better model fit.

### Prediction of gambling choice

Feeling values from Feeling Model 3 (found to be the most parsimonious model overall) were then used to predict choices in the gambling task. Specifically, the feeling associated with each amount was calculated from Feeling Model 3 using each participant’s estimated parameters (β and ρ). Thus, for each trial of the gambling task, a feelings value was obtained for the sure option, the gain, and the loss presented on that trial. A feelings value of 0 was used when the amount in the gambling trial was £0. The probability of choosing the gamble on each trial, coded as 1 if the gamble was chosen and 0 if the sure option was chosen, was then entered as the dependent variable of a logistic regression (choice model), with feelings associated with the sure option (*S*, coded negatively in order to obtain a positive weight), the gain (*G*, multiplied by its probability .5), and the loss (*L*, multiplied by its probability .5) entered as the three predictor variables:

$$p\left(\mathrm{gamble}\right)=\frac{1}{1+e^{–[\omega_{S}F\left(S\right)+\omega_{G}F\left(G\right)+\omega_{L}F\left(L\right)]}},$$

where ω is a weight value. For example, if a participant were offered a mixed-gamble trial in which he or she could choose either a gamble that offered a 50% chance of winning £10 and a 50% chance of losing £6 or a sure option of £0, we estimated the feelings associated with these three elements multiplied by their probability: the feeling associated with gaining £10, *F*(₤10) = β × 10^ρ^ × .5; the feeling associated with losing £6, *F*(–₤6) = β × (–6)^ρ^ × .5, and the feeling associated with getting £0: *F*(₤0) = 0 × 1 = 0.

Logistic regressions were run in MATLAB using the glmfit function, using either expected feelings (Choice Model 1) or experienced feelings (Choice Model 2). Each logistic regression resulted in three weight parameters ω, which reflected the weight assigned to feelings when making a choice: one for gains (ω_*G*_), one for losses (ω_*L*_), and one for sure options (ω_*S*_). To determine whether those modeled feelings predicted choice better than value-based models, we defined five other comparison models. One predicted choice from objective values (Choice Model 3), and another predicted choice from the log of objective values (consistent with standard economics models to account for the curvature of utility—Choice Model 4). The final three models were derived from prospect theory; in these models, value was weighted for each participant with his or her loss-aversion parameter (Choice Model 5), risk-aversion parameter (Choice Model 6), or both (Choice Model 7; see the Supplemental Material for more details). To avoid circularity and ensure all choice models were run on the same set of choice data, we estimated loss- and risk-aversion parameters using half the choice data; then, all seven choice models, including those in which we used extracted feelings rather than values, were run on the other half of the choice data.

To compare across conditions and participants, we standardized weight values ω using the following equation (Menard, 2004; Schielzeth, 2010):

$$\omega_{x}^{\prime }=\omega_{x}\frac{s_{x}}{s_{y}}$$

where $\omega_{x}^{\prime }$ is the standardized weight value, ω_*x*_ the original weight for predictor variable $\omega_{x}$ obtained from the regression, *s_x_* the standard deviation of variable *x*, and *s_y_* the standard deviation of the dependent variable *y*, here the binary choice values. Standardized weight values were extracted from each regression and compared using repeated measures analysis of variance (ANOVA) and paired-samples *t* tests.

### Replication and extension studies

Two separate studies were conducted to replicate the findings and extend them to cases in which the impact of a loss and a gain on feelings was evaluated (a) within the same trial (Replication and Extension Study 1) and (b) on the same unipolar rating scale (Replication and Extension Study 2). These studies suggest that the results are robust and not driven by these specific factors (see the Supplemental Material for details and results).

## Results

Our analysis followed two main steps. First, we used participants’ reported feelings associated with different monetary outcomes in the feelings task to build a feeling function. Specifically, we found the best-fitting computational model to specify how feelings associated with different amounts of gains and losses relate to the objective value of these amounts. Second, we tested whether that model of feelings predicted participants’ choices on the gambling task. Results of the main study are reported here, and results of the replication and extension studies are reported in the Supplemental Material.

### Characterizing a feeling function

For all the models described below, the method of computing change from the rating associated with the zero outcome (i.e., the rating associated with not winning or not losing the equivalent amount) resulted in the best fit (Table S1 in the Supplemental Material). Thus, we report results using this baseline; however, the results were the same when we used the other two methods of calculating feelings (see the Supplemental Material for details).

**Fig. 2.**
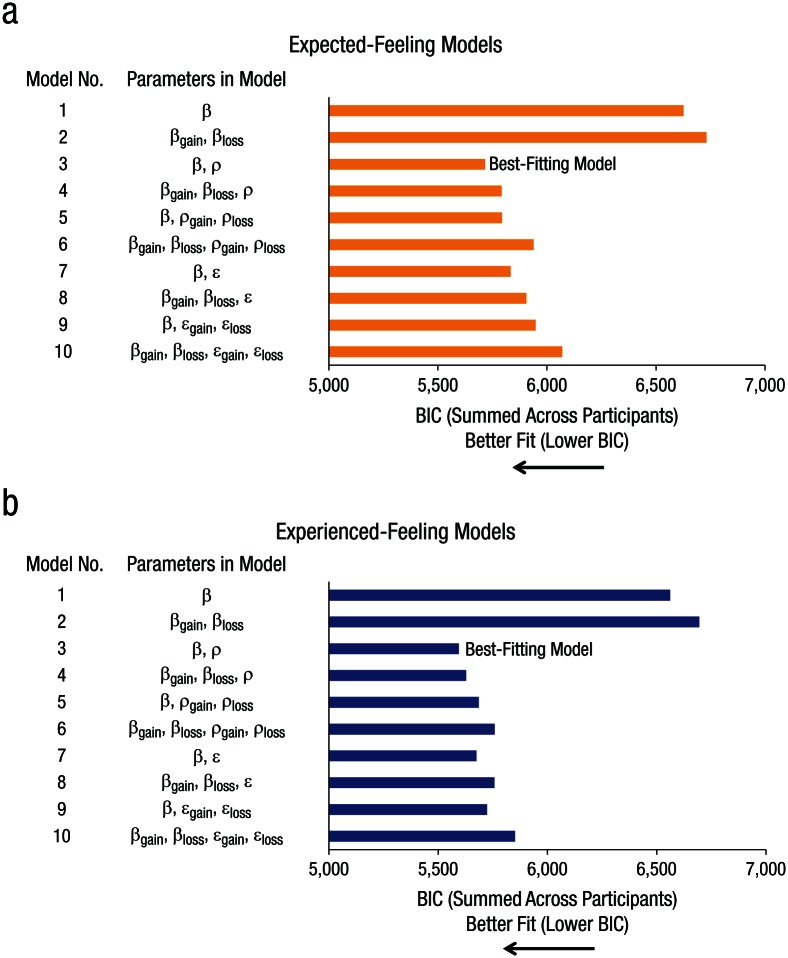
Bayesian information criterion (BIC), summed across participants, for each of the 10 models fitting feelings to outcome value, separately for (a) expected-feelings ratings and (b) experienced-feelings ratings.

**Fig. 3.**
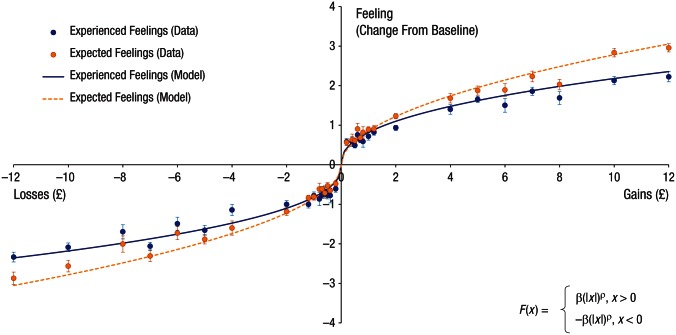
Feeling function. Plotted are expected- and experienced-feelings ratings averaged across participants for each outcome value, as well as predictions of the best-fitting model (Feeling Model 3). Feelings ratings are shown as a function of change from baseline (i.e., the rating associated with the zero outcome of neither winning nor losing). Error bars represent ±1 *SEM*.

The BIC, which penalizes for additional parameters, showed that the best-fitting model (i.e., the one with the lowest BIC value) for both expected (Fig. 2a) and experienced (Fig. 2b) feelings was Feeling Model 3 (see Table S2 in the Supplemental Material for BIC and *R*^2^ values), which has one ρ and one β. This suggests two things. First, it indicates that feelings’ sensitivity to outcomes gradually decreased as outcomes increased. Similar to the value function in prospect theory, ρ was significantly smaller than 1—expected feelings: ρ = .512, *SD* = .26, 95% confidence interval (CI) = [.418, .558], *t*(55) = −14.05, *p* < .001, Cohen’s *d* = 1.88; experienced feelings: ρ = .425, *SD* = .23, 95% CI = [.513, .637], *t*(55) = −18.52, *p* < .001, Cohen’s *d* = 2.5—which indicates that the feeling function was concave in the gain domain and convex in the loss domain. Figure 3 shows that the magnitude of feelings associated with £10, for example, was less than twice the magnitude of feelings associated with £5. The average β across participants, which represents the slope of the function, was 0.857 (*SD* = 0.36) for expected feelings and 0.819 (*SD* = 0.37) for experienced feelings.

Second, we found that neither sensitivity (β) nor curvature (ρ) differed for gains and losses. Equal sensitivity suggests that when feelings associated with losses and gains are evaluated separately, their impact is symmetrical, such that losses are not experienced more intensely than gains. On the surface, these findings contradict the notion of loss aversion, as proposed by prospect theory. However, what we will show later is that while here losses do not necessarily affect feelings more than gains, they are weighted to a greater extent when making a choice. With regards to curvature, a single ρ was more parsimonious than two separate ones for gains and losses, which suggests that the extent of concavity for gains was equivalent to the extent of convexity for losses.

Further support for the observation that feelings’ sensitivity to outcomes gradually decreased as outcomes increased came from the fact that all models with a curvature parameter ρ (Feeling Models 3–6) were better fits, as indicated by lower BIC values, than corresponding linear models with an intercept (Feeling Models 7–10). This was true both when comparing BICs for models fitting expected feelings (BIC difference < −112) and experienced feelings (BIC difference < −37; Table S2). Further support for the observation that neither sensitivity nor curvature differed between gains and losses came from the fact that Feeling Model 3 had lower BICs than other curved functions with additional parameters that fit gains and losses with separate parameters (Feeling Models 4–6; see Table S3 in the Supplemental Material) for both expected and experienced feelings. In addition, the absolute impact of losses and gains on ratings of feelings relative to a zero outcome revealed no difference, *F*(1, 55) = 0.01, *p* = .92, η_*p*_^2^ = .00018.

### Impact bias increases with the amount at stake

Interestingly, comparing the functions for experienced and expected feelings revealed an impact bias that increased with the amounts lost or gained. The impact bias is the tendency to expect losses or gains to affect feelings more than they actually do (Gilbert, Pinel, Wilson, Blumberg, & Wheatley, 1998). Specifically, the curvature (ρ) was smaller for the experienced-feeling function relative to the expected-feeling function—paired-samples *t*(55) = 3.31, *p* = .002, Cohen’s *d* = 0.442, 95% CI = [0.034, 0.138], while there was no difference in sensitivity values (βs), *t*(55) = 0.65, *p* = .52, Cohen’s *d* = 0.087, 95% CI = [−0.079, 0.155]. Thus, although both expected and experienced feelings became less sensitive to outcomes as absolute values of loss and gain increased, this diminished sensitivity was more pronounced in experience than in expectation. As a result, for small amounts of money gained or lost, people’s expectations of how they would feel were more likely to align with their experience. However, as amounts gained or lost increased, people were more likely to overestimate the effect of outcomes on their feelings, expecting to be affected more by gains and losses than they actually were (i.e., the impact bias; Gilbert et al., 1998). The growth of the impact bias can be seen in Fig. 3 as the increase in separation between the solid line (experienced feelings) and the more extreme (i.e., higher for gains, lower for losses) dashed line (expected feelings).

### Feeling function predicts choice better than value-based models

Once we established a function that fit feelings to outcome value, we turned to the question of how well those feelings predict choices, in particular how they are combined and weighted to make a decision.

**Fig. 4.**
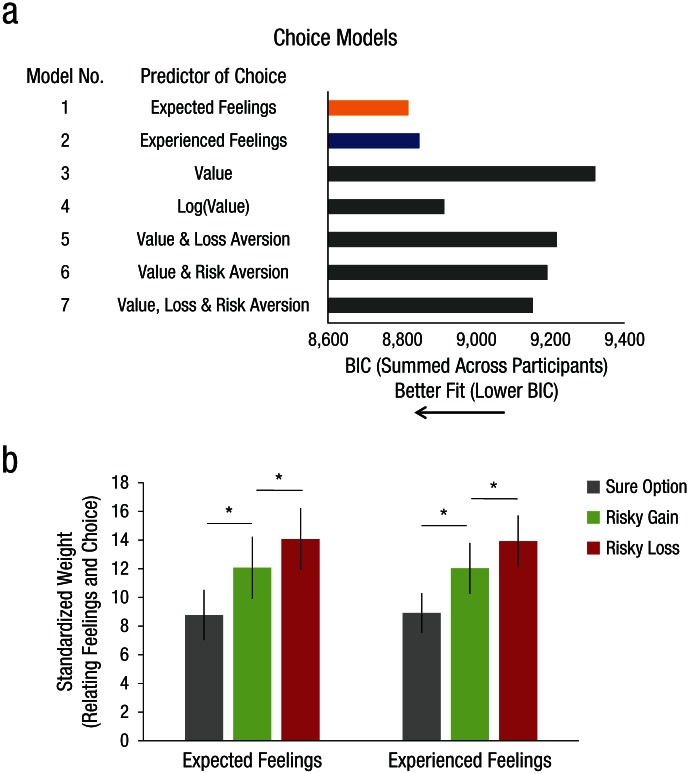
Results from the choice models. The Bayesian information criterion (BIC), summed across participants, is shown in (a) for each of the seven models predicting choices on the gambling task. Standardized weight parameters predicting choices from feelings are shown in (b) for each option of the gambling task, separately for expected feelings and experienced feelings (modeled from the feelings task). Higher values on the *y*-axis indicate that feelings about the corresponding gamble option (e.g., risky loss) are weighted more strongly during the decision. Error bars represent ±1 *SEM*. Asterisks represent significant differences between the options (*p* < .05), as determined by two-tailed paired-samples *t* tests.

Choice Models 1 and 2, in which choice was predicted from feelings extracted from the expected- and experienced-feeling function, respectively, predicted choice better than all value-based comparison models (Choice Models 3–7), as indicated by lower BIC scores (Fig. 4a) and higher *R*^2^ values (Table S4 in the Supplemental Material). Running the split-half analysis 100 times, with a different way to split the data on every iteration, revealed that models using feelings predicted choice better than all five comparison models in 99 iterations out of 100, thus confirming the reliability of this finding.

### Feelings associated with losses are weighted more than feelings associated with gains when decisions are being made

Are feelings about potential losses and gains given equal weights when people deliberate on a decision? Our feeling function indicated that the impact of a loss on feelings was equal to the impact of an equivalent gain. Yet while losses and gains may affect explicit feelings similarly, we found that these feelings are weighted differently when people are making a choice.

Specifically, ω parameters from our choice models, which predicted choices from feelings, revealed a greater weight for feelings associated with losses (ω_*L*_) relative to feelings associated with gains (ω_*G*_ ) in predicting choice—for expected feelings: *t*(55) = 3.04, *p* = .004, 95% CI = [0.684, 3.33], Cohen’s *d* = 0.406; for experienced feelings: *t*(55) = 2.93, *p* = .005, 95% CI = [0.599, 3.19], Cohen’s *d* = 0.392 (Fig. 4b). Models that allowed different weights for losses and gains performed significantly better than models that did not (Table S5 in the Supplemental Material).

Follow-up analysis revealed that this was true only in mixed-gamble trials, in which losses and gains are weighted simultaneously, but not in gain-only and loss-only trials, in which gains and losses are evaluated at different time points (different trials). Specifically, we ran logistic regressions to predict choice from feelings separately for each trial type, and then entered weight-of-feelings parameters into a 2 (trial type: mixed, nonmixed) by 2 (outcome: loss, gain) repeated measures ANOVA. This revealed a significant interaction—expected feelings: *F*(1, 55) = 6.54, *p* = .013, η_*p*_^2^ = .106; experienced feelings: *F*(1, 55) = 7.46, *p* = .008, η_*p*_^2^ = .119 (Fig. S1 in the Supplemental Material)—driven by a greater weight put on feelings associated with losses relative to feelings associated with gains during mixed-gamble choices—expected feelings: *t*(55) = 3.66, *p* = .001, 95% CI = [1.67, 5.71], Cohen’s *d* = 0.489; experienced feelings: *t*(55) = 2.45, *p* = .018, 95% CI = [0.91, 9.10], Cohen’s *d* = 0.327—but not during loss- versus gain-only trials—expected feelings: *t*(55) = 0.82, *p* = .42, 95% CI = [−3.25, 7.71], Cohen’s *d* = 0.109; experienced feelings: *t*(55) = 0.79, *p* = .43, 95% CI = [−2.75, 6.32], Cohen’s *d* = 0.105. In other words, only when potential losses and gains are evaluated simultaneously (i.e., in the same choice) are feelings about losses weighted more strongly than feelings about gains. Results of our first replication and extension study further show that even when gains and losses were evaluated in the same trial during the feelings task, their impact on feelings does not differ, but their weight on gambling choices does (see the Supplemental Material for details).

To further tease apart the asymmetrical use of feelings associated with gains and losses in shaping choice from the use of value alone, we ran another logistic regression (Choice Model 8) in which raw feelings (i.e., reported feelings relative to baseline rather than those derived from the feeling function) were added as predictors of choice in the same logistic regression as objective values themselves. This was done to reveal the weight assigned to feelings in making a choice over and beyond the effect of value per se, when the two compete. The results showed no difference in the weight assigned to the value of losses and gains per se, *t*(55) < 1.2, *p* > .23, Cohen’s *d* < .17, only to the weight assigned to the associated feelings—expected feelings: *t*(55) = 3.59, *p* = .001, 95% CI = [1.29, 4.55], Cohen’s *d* = .479; experienced feelings: *t*(55) = 2.28, *p* = .027, 95% CI = [0.197, 2.89], Cohen’s *d* = 0.307. Again, this was true only for mixed-gamble choices, not for gain-only or loss-only trials, in which neither feelings nor values were weighted differently between losses and gains (Table S6 in the Supplemental Material). This suggests that losses are not weighted differently from gains; rather, *feelings* associated with losses and with gains are weighted differently, which emphasizes the importance of feelings in decision making.

**Fig. 5.**
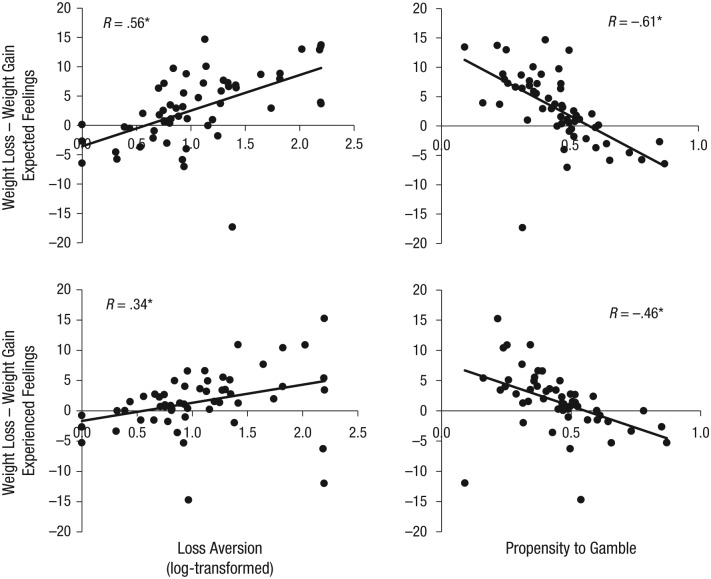
Relation between individual choice behavior and weight difference in feelings associated with losses and gains. The left column shows correlations between loss aversion and the loss-gain weight difference for expected feelings (top) and experienced feelings (bottom). The right column shows correlations between the propensity to gamble and loss-gain weight difference for expected feelings (top) and experienced feelings (bottom). Best-fitting regression lines are shown for each set of data. Asterisks indicate that linear correlations were significant (*p* < .05). These correlations indicate that the greater weight a participant put on feelings associated with a loss relative to a gain when making a decision, the more loss averse (and less likely to gamble) they were.

This last conclusion raises the possibility that individual differences in decision making could be explained by how people weigh feelings when making a choice. Indeed, using the weights from Choice Model 8, we found that, controlling for value, individual differences in both loss aversion and the propensity to choose gambles were directly correlated with the extent to which feelings associated with losses were overweighted compared with feelings associated with gains—correlation between loss aversion and loss-gain weight difference for expected feelings: *r*(56) = .56, *p* < .001; for experienced feelings: *r*(56) = .34, *p* = .012; correlation between propensity to gamble and loss-gain weight difference for expected feelings: *r*(56) = −.61, *p* < .001; for experienced feelings: *r*(56) = −.46, *p* < .001 (Fig. 5; see the Supplemental Material for loss-aversion modeling). Specifically, participants who weighted feelings associated with losses more than feelings associated with gains were more loss averse and less likely to gamble.

This set of results suggests that the asymmetric influence of gains and losses on decision making, as suggested by prospect theory, is not necessarily reflected in expected or experienced feelings, or in different weights assigned to value per se, but rather in the extent to which feelings associated with losses and gains are taken into account when one makes a decision.

## Discussion

The relationship between people’s feelings and the choices they make has occupied scientists, policymakers, and philosophers for decades. Indeed, in recent years, numerous studies have investigated how decisions and outcomes affect people’s feelings (Carter & McBride, 2013; Kassam et al., 2011; Kermer et al., 2006; McGraw et al., 2010; Mellers et al., 1997; Rutledge et al., 2014; Yechiam et al., 2014) and life satisfaction (Boyce, Wood, Banks, Clark, & Brown, 2013; De Neve et al., 2015). Yet the equally critical question of how people’s explicit feelings affect their decisions has been relatively neglected. In this study, we addressed this important question in a controlled laboratory setting and modeled how feelings are integrated into decisions. We demonstrated that feelings drive the decisions people make. However, the rules by which they do so differ from those that were previously assumed.

Our feeling model predicted choice better than objective values did, and a unique contribution of feelings in the decision process was demonstrated. The feeling function that best related feelings to value was revealed to be concave for gains and convex for losses, much as the value function in prospect theory (Kahneman & Tversky, 1979; Tversky & Kahneman, 1992) and other nonlinear utility functions (Bernoulli, 1954; Fox & Poldrack, 2014; Stauffer, Lak, & Schultz, 2014; Von Neumann & Morgenstern, 1947). This curvature suggests that explicit feelings, similar to subjective value or utility, show diminishing sensitivity to outcomes as the value of these outcomes increases (Carter & McBride, 2013). In other words, when it comes to one’s feelings, the impact of winning or losing $10 is less than twice that of winning or losing $5.

Our feeling model also revealed no asymmetry between gains and losses, which suggests that the impact of a loss on feelings is not necessarily greater than the impact of an equivalent gain. This finding was replicated in two additional studies in which a gain and a loss were evaluated at the same time and in which the associated feelings about gains and losses were reported using the same unipolar scale. We do not suggest that the feelings associated with losses and gains will always be symmetric. On the contrary, different stimuli and contexts may result in varying asymmetric effects (Harinck et al., 2007; McGraw et al., 2010). In particular, in contrast to the findings reported here, a loss-gain asymmetry in feelings has been previously reported in a study using a one-shot game, in which the stakes consisted of large ($200) hypothetical amounts (McGraw et al., 2010). Our study examined responses to incentive-compatible, but relatively small, gains and losses. It is possible that for higher amounts, an asymmetry in feelings would emerge. However, we speculate that even for large stakes, the feeling function may do a better job at predicting choice than value alone. That question awaits testing.

Despite this absence of asymmetry in feelings, we found that loss aversion was still present in choice (see the Supplemental Material for the mean and median statistics for loss aversion as well as details on the proportions of risky choices), consistent with the predictions of prospect theory. Importantly, when participants made a decision, a greater weight was put on feelings associated with losses relative to gains. Therefore, our finding suggests that even when losses do not affect feelings more strongly than gains do, those feelings are weighted more when making a choice than feelings about gains. Moreover, the amount by which feelings associated with losses are overweighted relative to feelings associated with gains when one makes a decision relates to individual differences in loss aversion and propensity to gamble.

This finding resolves a long-standing puzzle in which loss aversion is often observed in choice but not necessarily in explicit feelings (Harinck et al., 2007; Kermer et al., 2006; McGraw et al., 2010; Mellers et al., 1997). We suggest that the asymmetric influence of gains and losses on decision making, as suggested by prospect theory, is “reflected neither in expected or experienced feelings directly, nor in different weights assigned to value per se, but” in the extent to which feelings about losses and gains are taken into account when people make a decision. Our result is consistent with the interpretation of an increased attention to losses when one makes a choice (Yechiam & Hochman, 2013). When losses and gains are presented separately in a decision, the feelings associated with them are weighted in a symmetrical way. However, when they compete for attention, as is the case in mixed gambles, people may allocate more attention to the feelings they would derive from the loss than from the gain, which leads them to choose in a loss-averse manner. It is also possible that people implicitly experience losses to a greater extent than they experience gains (Hochman & Yechiam, 2011; Sokol-Hessner et al., 2009), but this difference is not exhibited in explicit reports.

Our findings also provide the first demonstration of an impact bias that increases with value. Specifically, we found that participants’ self-report feelings exhibited an impact bias (also called affective-forecasting error), such that they expected the emotional impact of an event to be greater than it actually turned out to be (Gilbert et al., 1998; Kermer et al., 2006; Kwong, Wong, & Tang, 2013; Levine, Lench, Kaplan, & Safer, 2013; Morewedge & Buechel, 2013; Wilson & Gilbert, 2013). Interestingly, this impact bias was not constant but increased with value. This was because of a stronger curvature of experienced feelings relative to expected feelings. In other words, as absolute value increased, sensitivity to value diminished more quickly for experienced relative to expected feelings. This suggests that as people win or lose more money, they are more and more biased toward overestimating the emotional impact of these outcomes.

Our modeling approach provides novel insight into how explicit feelings relate to choice. Such understanding is of theoretical importance and also has practical implications for policymakers, economists, and clinicians who often measure explicit feelings to predict choice (Benjamin, Heffetz, Kimball, & Rees-Jones, 2012, 2014).

## Supplementary Material

Supplementary material
