# Supplementary material for: Models of Affective Decision Making: How Do Feelings Predict Choice?
Source: Psychol Sci. 2016 Apr 12;27(6):763–75. doi: 10.1177/0956797616634654 (PMC4904352; doi:10.1177/0956797616634654)
Supplement: Supplementary material [file suppl-material.pdf]

DOI: 10.1177/0956797616634654

Supplemental Material

Models of affective decision making: How do feelings predict choice?

Supplemental Methods and Results

Study groups

Participants were recruited in two different groups that were then collapsed in the analyses. A group of 29 participants (20 females, mean age=23.2y) were tested on a first version of the task, where each of the four blocks had 48 trials with different amounts (£0.2, £0.4, £0.6, £0.8, £1, £1.2, £2, £4, £6, £8, £10, £12) that could be won, lost, not won or not lost. For expected feelings participants were asked "how will you feel if you win/lose?"; and for experienced feelings "how do you feel now?". The rating scale ranged from 1 (extremely unhappy) to 10 (extremely happy) and participants had to press a key (1 to 9 for ratings 1 to 9 and 0 for rating 10) to indicate their feelings. A second group of 30 participants (15 females, mean age=24.5y) completed a slightly shorter version of the feelings task that had 40 trials per block (10 amounts instead of 12: £0.2, £0.5, £0.7, £1, £1.2, £2, £5, £7, £10, £12) and indicated their ratings by moving a cursor on a symmetrical rating scale, in which 0 was used as a reference point. Specifically, for expected feelings they were asked "if 0 is how you feel now, how will you feel if you win/lose?"; and for experienced feelings "if 0 is how you felt just before the choice, how do you feel now?". Ratings ranged from -5 (extremely less happy) to +5 (extremely more happy). The first group of participants completed the feelings task first, while the second group completed the gambling task first. The data (parameters and model fits from the feelings function models, and from the regression models to predict choice) did not differ between the two study groups, indicating that those features of the design that varied between the two groups were not a significant factor. Data were therefore collapsed for all the analyses reported in the main text, and study group was controlled for by adding a dummy variable as a between-subject factor in all the analyses.

DOI: 10.1177/0956797616634654

Agency manipulation in the feelings task

The instrumental choice present in the feelings task (i.e. the arbitrary selection between the two abstract stimuli) allowed us to manipulate agency: on 2 of the blocks (1 with expected feelings and 1 with experienced feelings) the participant made the choice between the two stimuli, and in the other 2 the computer made the choice for the participant who had to indicate the computer choice with a button press after it was made. There were no differences in the data between own choice and computer choice blocks, therefore data was collapsed. Even when making their own choices subjects had no control over the outcome, thus it may not be surprising that feelings did not differ between own choice and computer choice. Note, that the above relates only to the task in which we elicited feelings associated with outcomes and not, obviously, to the gambling task.

Estimation of Feeling Models

All ten Feeling Models were estimated using a maximum-likelihood estimation procedure in Matlab. Given a Feeling Model $f(x,\theta)$ with θ the set of parameters, x the range of outcome values, and y the feelings data to be modeled, the residuals from the model can be written as:

$$\mathcal{E} = y - f(x,\theta) \qquad \text{(Eq. S1)}$$

Assuming an appropriate normal distribution for the residuals, the likelihood of a given residual $\mathcal{E}_i$ is:

$$\mathcal{L}(\mathcal{E}_i|\theta,\sigma) = \frac{e^{\frac{-\mathcal{E}_i}{2\sigma^2}}}{\sqrt{2\pi\sigma^2}} \qquad \text{(Eq. S2)}$$

where σ represents the standard deviation of the residuals (an additional parameter to be estimated). Then the fmincon function was used to find the optimal set of parameters (θ, σ) that minimizes the negative log likelihood (thereby maximizing the likelihood):

$$-\log \mathcal{L} = -\log[\mathcal{L}(\mathcal{E}|\theta,\sigma)] = \sum_i \left[\frac{\mathcal{E}_i^2}{2\sigma^2} + 0.5\log(2\pi\sigma^2)\right] \qquad \text{(Eq. S3)}$$

BIC scores were then calculated for each subject using the following equation that penalizes additional parameters in the model:

DOI: 10.1177/0956797616634654

$$BIC = -2\log\hat{\mathcal{L}} + k\log(n) \quad \text{(Eq. S4)}$$

where $\log\hat{\mathcal{L}}$ represents the maximum of loglikelihood $\mathcal{L}$ (estimated using equation S3 above), k the number of parameters in the model (including σ as an extra parameter), and n the number of data points (trials) that were fitted.

Loss and risk aversion modelling

In order to assess loss and risk aversion, three models were estimated for each subject using choice data from the gambling task and based on Prospect Theory equations (Fox & Poldrack, 2014; Kahneman & Tversky, 1979; Sokol-Hessner et al., 2009). The model was estimated in Matlab using a maximum likelihood estimation procedure. For each trial, the utility (u) of each gamble was estimated using one of the three following equations:

$$u(gamble) = 0.5 \times gain + 0.5 \times \lambda \times loss \quad \text{(Eq. S5 - to estimate loss aversion only)}$$

$$u(gamble) = 0.5 \times gain^{\gamma} + 0.5 \times loss^{\gamma} \quad \text{(Eq. S6 - to estimate risk aversion only)}$$

$$u(gamble) = 0.5 \times gain^{\gamma} + 0.5 \times \lambda \times loss^{\gamma} \quad \text{(Eq. S7 – loss and risk aversion together)}$$

where λ is the "loss aversion" parameter: a λ value higher than 1 indicates an overweighing of gains relative to losses during decision-making and a λ value lower than 1 the converse; and γ is the "risk aversion" parameter: a γ value lower than 1 indicates diminishing sensitivity to changes in value and results in risk aversion, while a γ value higher than 1 indicates risk-seeking.

These utility values were used in a softmax function to estimate the probability of accepting each gamble (coded as 0 or 1 for each rejected or accepted gamble, respectively):

$$P(gamble) = \frac{1}{1+e^{-\mu \times u(gamble)}} \quad \text{(Eq. S8)}$$

where μ is the logit sensitivity or "inverse temperature" parameter, an index of choice consistency for repeated identical gambles, equivalent to the maximal slope of a logistic regression curve: higher μ values indicate more consistent choices.

The three models were used to estimate risk and loss aversion on half the choice data, in order to predict choice from subjective utility on the other half of choice data (see "Comparison models to predict choice" paragraph below).

To predict individual differences in loss aversion from feelings, λ values were extracted for each subject on the entire set of gambling choices using equation S5. They were then log-transformed [ln(λ+1)] to ensure positive values and normal distributions, and correlated across subjects with the difference in how feelings about losses and feelings about gains are weighted during choice (Fig. 5A-B).

Across all participants in the main study, mean loss aversion (λ) was 2.38 (±SD=2.19), significantly greater than 1 ($t(55)=4.72$, $P<0.001$, Cohen's $d_z=0.63$, 95% CI=[1.80;2.97]). In the replication and extension studies, mean loss aversion was 2.49 (±SD=2.10, $t(19)=3.17$, $P=0.005$, Cohen's $d_z=0.71$, 95% CI=[1.51;3.48]) and 2.38 (±SD=2.14, $t(29)=3.54$, $P=0.001$, Cohen's $d_z=0.65$, 95% CI=[1.58;3.18]) for study 1 and 2, respectively. However, because the distribution of this loss aversion parameter is often positively skewed, an average parameter greater than 1 may be driven by few highly loss averse subjects; therefore, we also calculated the median loss aversion to ensure it was also above 1 (similar to Tom, Fox, Trepel, & Poldrack, 2007). This was the case in all three studies, with median λ values of 1.64, 2.17 and 1.47 for the main study, replication and extension studies 1 and 2, respectively. Finally, gambling choices on mixed gamble trials can also be indicative of loss aversion. To investigate this, we calculated for each study group the proportion of risky choice for each gamble across all subjects. Results are shown in Figure S2 and reveal that participants reliably avoid gambling when the loss and gain amounts are the same (shown by a proportion of risky choice between 20-40% along the diagonal of the matrices in Fig. S2). Across all studies their indifference point (gambles for which the proportion of risky choice is 50%) is shifted towards gambles with a positive expected value (i.e. gain value>loss value), such that the gain value needs to be about twice as big as the loss value for participants to start gambling more than 50% of the time. This indicates that loss aversion was reliably present in choice and across the whole range of gambles.

Methods of computing feelings

The impact of losses and gains on feelings were computed using three different methods: as the change from the mid-point of the rating scale, as the change from the previous rating, and

DOI: 10.1177/0956797616634654

as the change from the rating associated with zero outcome (i.e., the rating associated with not winning or not losing the equivalent amount). For all ten Feelings Models the latter baseline resulted in the best fit (Table S1), which is why we report results using this baseline in the main text. However, we note that results were the same when using the other two methods of calculating feelings. First, when we estimated Choice Models to predict gambling choice from these feelings functions varying in their reference point, we replicated our finding that these feelings predicted choice better than the five other value-based Choice Models (Choice Model using expected feelings from scale mid-point: BIC=8884, R^2=0.30; Choice Model using experienced feelings from scale mid-point: BIC=8915, R^2=0.30; Choice Model using experienced feelings from previous trial feeling: BIC=8924, R^2=0.30; value-based Choice Models: BIC>9025, R^2<0.29). Second, we also find that feelings about losses are weighted more than feelings about gains in predicting choice, independent of the baseline used to calculate feelings (expected feelings from scale mid-point: t(55)=3.38, P=0.001; experienced feelings from scale mid-point: t(55)=3.33, P=0.002; experienced feelings from previous trial feeling: t(55)=3.20, P=0.002). This suggest that our findings do not depend on the method of calculating feelings.

Comparison models to predict choice

Choices were predicted from feelings using the previously built feelings function (Choice Models 1 and 2). In order to examine whether this feelings function does a better job at predicting choice than objective value, or choice-derived subjective utility, five other models were tested (Choice Models 3 to 7).

First a simple “Value” model (Choice Model 3) tries to predict choice simply by entering the amounts available multiplied by probability, regardless of associated feelings parameters β and ρ or subjective utility parameters such as loss and risk aversion. For example, if the choice is a mixed gamble between winning £10 and losing £6, the three predictors will be £0*1 (sure option), £10*0.5 (gain), and -£6*0.5 (loss).

The second comparison model included log(Value) as predictors (Choice Model 4). Most standard economic models account for the curvature of utility by taking the logarithm of linear values. In this model and with the example above, the three predictors would be computed as: 0 (sure option), log(10)*0.5 (gain), and -log(6)*0.5 (loss).

DOI: 10.1177/0956797616634654

The three additional models predicted choice from Prospect Theory-derived subjective utility. To do so, risk and loss aversion parameters were estimated on half the choice data using the model described above (equations S5 to S8) for each subject. One model included value weighted with the loss aversion parameter $\boldsymbol{\lambda}$ ($£0 \times 1$, $£10 \times 0.5$, $-\boldsymbol{\lambda} \times £6 \times 0.5$; Choice Model 5); one included value parameterized with the risk aversion parameter $\boldsymbol{\gamma}$ ($£0 \times 1$, $(£10)^{\gamma} \times 0.5$, $-(£6)^{\gamma} \times 0.5$; Choice Model 6); and the last model included both loss and risk aversion to compute subjective values ($£0 \times 1$, $(£10)^{\gamma} \times 0.5$, $-\boldsymbol{\lambda} \times (£6)^{\gamma} \times 0.5$; Choice Model 7).

All seven logistic regression choice models were run on the other half of the choice data, in order to be comparable and to avoid circularity for the utility-based models. The gambling task was designed such that each gamble was repeated twice; therefore, one occurrence of each gamble was present in each half of the data. In addition, in order to ensure the reliability of this split-half analysis, 100 simulations were run with a different data splitting on every simulation. The loglikelihood of each model was extracted from the logistic regression and BIC scores were calculated for each subject using equation S4. The sum of BIC scores across subjects was then calculated for each model and each simulation, therefore allowing us to report the number of simulations where the two feelings model performed better than the five comparison models.

Replication and extension study 1

Rationale. Because the feelings task reported in the main text elicits feeling ratings about gains and about losses on separate trials, this design does not rule out the possibility that losses and gains may impact feelings differently when they are evaluated at the same time.

Methods. Thus, a follow-up study was run using exactly the same procedure as before, except that on each trial of the feelings task (Fig. S3), the outcomes at stake included a gain, a loss, and £0 (rather than gain versus £0 on some trials, and loss versus £0 on different trials). Twenty participants were recruited and tested on this paradigm (12 males, 8 females, mean age = 23.8 years, age range = 19-33). Ten participants completed the feelings task first, and the remaining completed the gambling task first. Block order within the feelings task was also counterbalanced across subjects. The range of amounts and rating scale used were the same as in the second study group of the main study (see *"Study groups"* paragraph above). Participants were told that each picture from the pair was associated with a certain probability

DOI: 10.1177/0956797616634654

to win, lose, or get £0, and that these probabilities were different for each picture and not shown to them. Therefore participants had to rate their feelings on every trial knowing that each picture chosen could result in a gain, a loss, or a null outcome (£0). To maintain consistency with the previous design, participants were only asked to rate their expected feelings about 2 of the 3 potential outcomes on each trial. These were determined such that each amount from £0.2 to £12 (win or lose) had at least one expected feeling rating associated with it; then the other rating was selected randomly from the other two options. The order of the two ratings was randomized. The impact of losses and gains on feelings were computed using three different baselines as in the main experiment. For all ten feelings models, using the change from the mid-point of the rating scale resulted in best fit of both expected and experienced feelings data as indicated by higher R^2 values and lower BIC values, and was therefore used for all the analyses below. Note that in contrast with the main experiment the zero baseline did not result in the best fit of feelings data. This is because in the replication study the zero outcome was always associated with two possible outcomes instead of one. Thus, the zero baseline was calculated differently – for each amount (for example £2), the ratings associated with £0 were averaged across all trials where that specific amount (£2) was at stake, regardless of third amount presented (which could be for example -£1, or -£10) – this conceptually and mathematically different approach resulted in different model fits.

Results

Feeling Models. Feelings were fit with the ten Feeling Models described in the main Methods to determine which function best relates feelings to value. If gains and losses impact feelings differently when evaluated at the same time, then a Feeling Model with different parameters (for example, a different slope β) for gains and losses, such as Feeling Model 4 or 6, should fit the feelings data better. However, this was not the case; instead we replicated our previous finding showing that Feeling Model 3, with a single slope (β) and single curvature (ρ) parameter for gains and losses, was the most parsimonious function that explains how feelings relate to value (Fig. S4). This result replicates our previous finding that gains and losses impact feelings similarly and extends to cases where the loss and the gain are evaluated together.

Choice Models. To examine whether and how these feelings are weighed to predict choice, feelings extracted from best fitting Feelings Model 3 were entered in a logistic regression to predict choice on the gambling task. The same seven Choice Models were run as in the main

data, again replicating our finding that feelings predicted choice better than value-based models (as indicated by lower BIC scores and higher R^2 values for Choice Models 1 and 2; Fig. S5A). Importantly, during choice, participants also weighed their feelings about losses more than their feelings about gains (expected feelings: t(19)=2.41, *P*=0.027; experienced feelings: t(19)=2.32, *P*=0.032; Fig. S5B). Finally, the extent to which feelings about losses were weighed more than feelings about gains (in a separate Choice Model controlling for the effect of value) was positively associated with individual estimates of behavioral loss aversion (expected feelings: r(20)=0.54, *P*=0.014; experienced feelings: r(20)=0.44, *P*=0.052; Fig. S5C).

Replication and extension study 2

Rationale. A recent study (McGraw, Larsen, Kahneman, & Schkade, 2010) has reported that measuring feelings on a bipolar scale, like we do in our main experiment, resulted in no gain/loss asymmetry in feelings, consistent with our findings, while using a unipolar scale (which represents the magnitude of feelings only) does result in an asymmetry. The suggestion is that a unipolar scale allows positive and negative feelings to be directly scaled relative to one another. We thus reran our experiment using a unipolar scale.

Methods. We collected data on an independent group of 30 participants (15 males, 15 females, mean age = 24 years, age range = 18-35). The procedure was the same as in the main study, except that a unipolar rating scale was used in the feelings task. A power analysis indicated that a sample size of 30 would give us 99% power to detect an effect size similar to the one observed in McGraw et al (d=0.76 for the difference between feelings for gains and losses using the unipolar scale) at a threshold of $p<0.05$. Even if the actual effect size is lower (d=0.5), achieved power would be 85%.

On experienced feelings trials the question was “How is this outcome affecting your feelings now?”. On expected feelings gain trials subjects were asked “How would winning £X affect your feelings?” and “How would not winning £X affect your feelings?”, and on expected feelings loss trials “How would losing £X affect your feelings?” and “How would not losing £X affect your feelings?”. Participants responded by moving a cursor on a scale ranging from 0 (“No effect”) to 5 (“Very large effect”). For analysis, ratings associated with losing and with not winning were coded negatively. Analysis then proceeded exactly as in the main experiment.

Results

DOI: 10.1177/0956797616634654

Feeling Models. As in the main experiment Feeling Model 3, with a single slope (β) and single curvature (ρ) parameter for gains and losses, was the best model of the ten in explaining how feelings relate to value (Fig. S6). This suggests that even when using the same unipolar scale that allows scaling positive and negative feelings relative to each other regardless of valence, gains and losses have a symmetrical impact on feelings.

Bayes Factor analysis. In addition, a Bayes Factor analysis was run on the feelings data using JASP (version 0.7.1; Love et al., 2015; Morey & Rouder, 2015). A Bayesian repeated-measures ANOVA was conducted with domain (gain/loss) and amount (the range of 10 amount values from £0.2 to £12) as within-subject factors. The winning Bayesian ANOVA model included a main effect of amount, but no effect of domain or domain*amount interaction, consistent with our Feeling Models result. In particular, adding a main effect of domain made the model about 11 times worse (BF[Amount Model over Amount & Domain Model]=10.87 for expected feelings and 10.62 for experienced feelings), offering strong evidence for an absence of feelings asymmetry between gains and losses (for correspondence between BF magnitude and strength of evidence, see Jarosz & Wiley, 2014; Jeffreys, 1961).

Choice Models. As in the main experiment, feelings extracted from best fitting Feeling Model 3 predicted choice better than value-based models (as indicated by lower BIC scores and higher R^2 values for Choice Models 1 and 2; Fig. S7A). During choice, we again find that participants weighted their feelings about losses more than their feelings about gains (expected feelings: $t(29)=2.29$, $P=0.030$; experienced feelings: $t(29)=2.08$, $P=0.047$; Fig. S7B). Finally, we also replicate our finding that the extent to which participant overweigh their feelings about losses relative to gains (in an additional Choice Model where the effect of value *per se* is accounted for) predict individual differences in behavioral loss aversion (expected feelings: $r(30)=0.62$, $P<0.001$; experienced feelings: $r(30)=0.63$, $P<0.001$; Fig. S7C).

With these additional studies, we replicate our findings in two further independent samples, thereby confirming and strengthening our results.

Supplemental References

Fox, C. R., & Poldrack, R. A. (2014). Prospect Theory and the brain. In *Handbook of Neuroeconomics (2nd edition)*. New York: Elsevier.

Jarosz, A. F., & Wiley, J. (2014). What are the odds? A practical guide to computing and reporting Bayes Factors. *Journal of Problem Solving*, *7*, 2–9. http://doi.org/http://dx.doi.org/10.7771/1932-6246.1167

Jeffreys, H. (1961). *Theory of probability* (3rd Ed.). Oxford, UK: Oxford University Press.

Kahneman, D., & Tversky, A. (1979). Prospect theory: an analysis of decision under risk. *Econometrica*, *47*(2), 263–292. http://doi.org/10.2307/1914185

Love, J., Selker, R., Marsman, M., Jamil, T., Dropmann, D., Verhagen, A. J., … Wagenmakers, E.-J. (2015). JASP (Version 0.7.1) [Computer Software].

McGraw, A. P., Larsen, J. T., Kahneman, D., & Schkade, D. (2010). Comparing gains and losses. *Psychol Sci*, *8*(6), 423–429. http://doi.org/10.1177/0956797610381504

Morey, R. D., & Rouder, J. N. (2015). BayesFactor (Version 0.9.11-3) [Computer Software].

Sokol-Hessner, P., Hsu, M., Curley, N. G., Delgado, M. R., Camerer, C. F., & Phelps, E. A. (2009). Thinking like a trader selectively reduces individuals' loss aversion. *Proc Natl Acad Sci U S A*, *106*(13), 5035–5040.

Tom, S. M., Fox, C. R., Trepel, C., & Poldrack, R. A. (2007). The neural basis of loss aversion in decision-making under risk. *Science*, *315*(5811), 515–8. http://doi.org/10.1126/science.1134239

Supplemental Figures and Tables

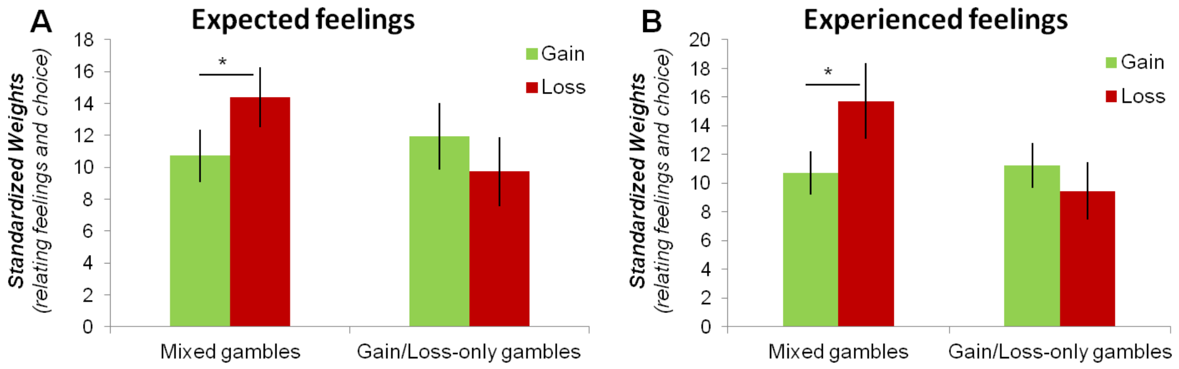

Fig. S1. Influence of gamble type on differential weighting of feelings associated with losses versus gains. Logistic regressions were run to predict choice from feelings separately for each trial type. Standardized parameter estimates representing the decision weight of feelings were analyzed in a two (trial type: mixed/non-mixed gambles) by two (outcome:

loss/gain) repeated-measures ANOVA. Significant interactions for both expected (**A**) and experienced (**B**) feelings indicate that more weight is given to feelings about a loss relative to a gain only when the loss and the gain are evaluated simultaneously (i.e. in the same gamble). Error bars denote SEM. Paired t-tests: * *P*<0.05.

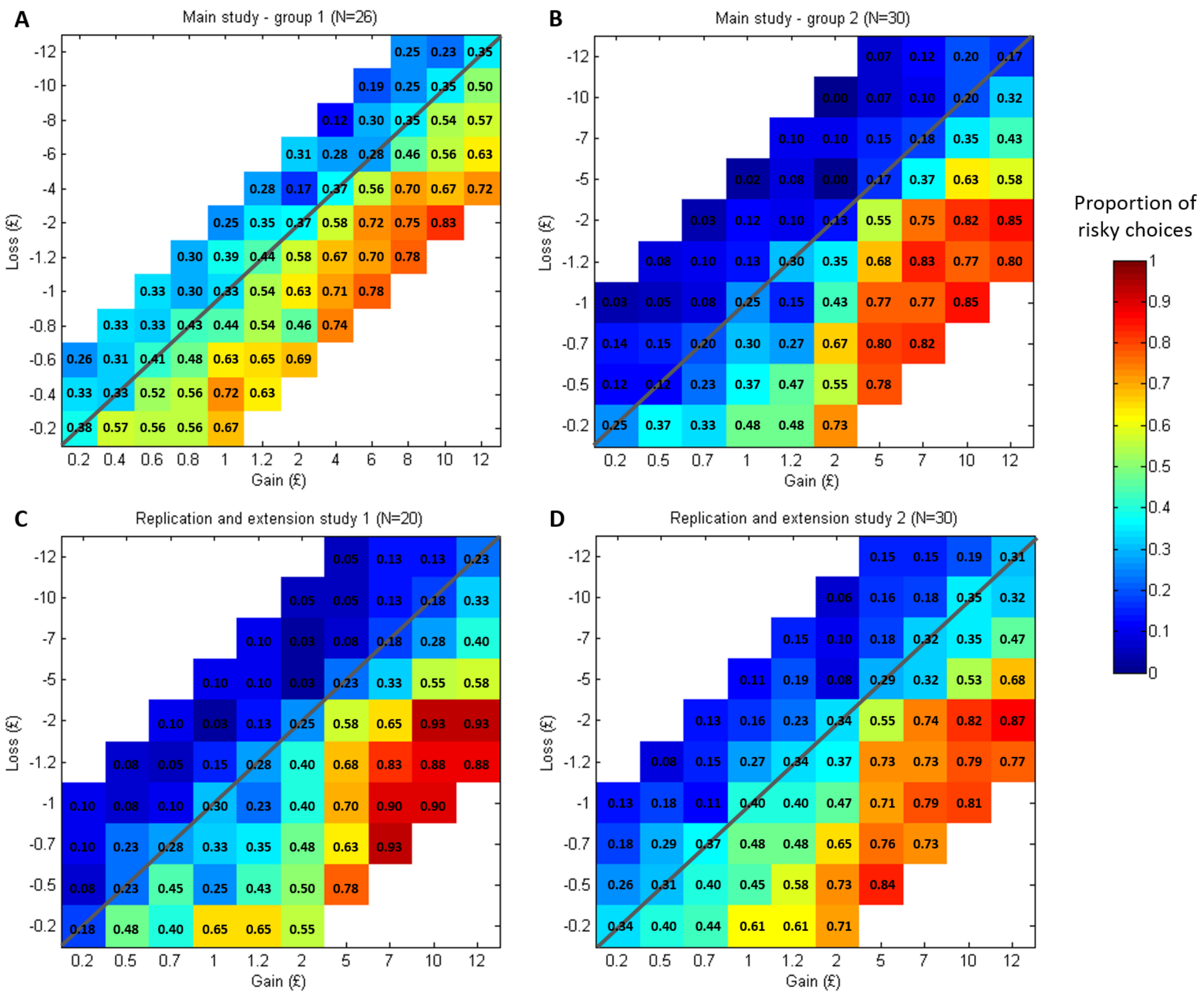

Fig. S2. Proportion of risky choices detailed for each mixed gamble trial from the choice task. Each table shows the gain and loss values used to create the range of mixed gambles. Each gamble constituted of 50% chance to win the gain amount and 50% chance to lose the loss amount. As detailed in the first paragraph of the Supplemental Material (‘Study groups’ paragraph), a slightly different range of values were used for the first (**A**) and second (**B**) groups of the main study, resulting in 71 and 69 gamble types, respectively. Both replication and extension studies (**C** and **D**) used the same gambles as **B**. Each gamble was presented twice. For each subject, the propensity of choosing each of these gambles over the sure option of £0 was calculated by averaging over the 2 trials where the specific gamble was shown, then the mean proportion of risky choices was calculated by averaging that probability across all subjects in each group. The number and color inside each cell both indicate that mean proportion of risky choices. Loss aversion is reflected in each group by reliable avoidance of gambles with the same gain and loss value (proportion of risky choices

DOI: 10.1177/0956797616634654

always <50% along the diagonal) and participants starting to choose the gamble more than 50% of the time only when the gain value was about twice as big as the loss value.

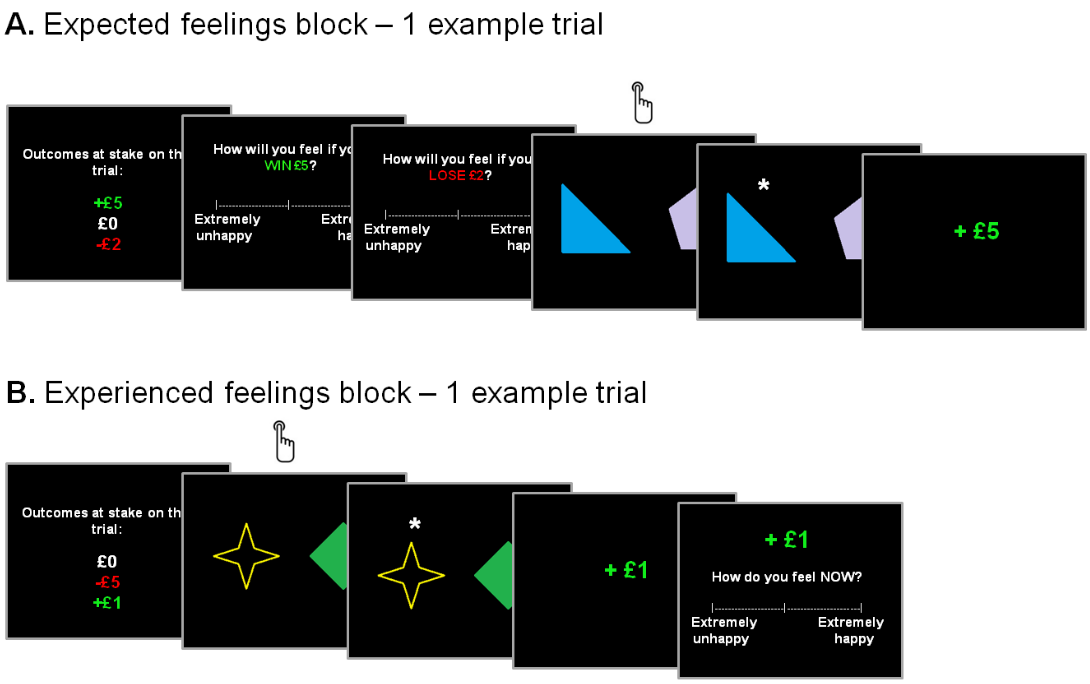

Fig. S3. Replication and extension study 1 – design of the feelings task. An additional study was run to replicate the finding and test whether gains and losses impact feelings differently when they are evaluated in the same trial. Task structure was similar to the main study (main text **Fig. 1**), except that on each trial of the feelings task, 3 potential outcomes were presented to the subject, always including a gain, a loss, and a null outcome (£0). The design of the gambling task (main text **Fig. 1C**) remained the same.

DOI: 10.1177/0956797616634654

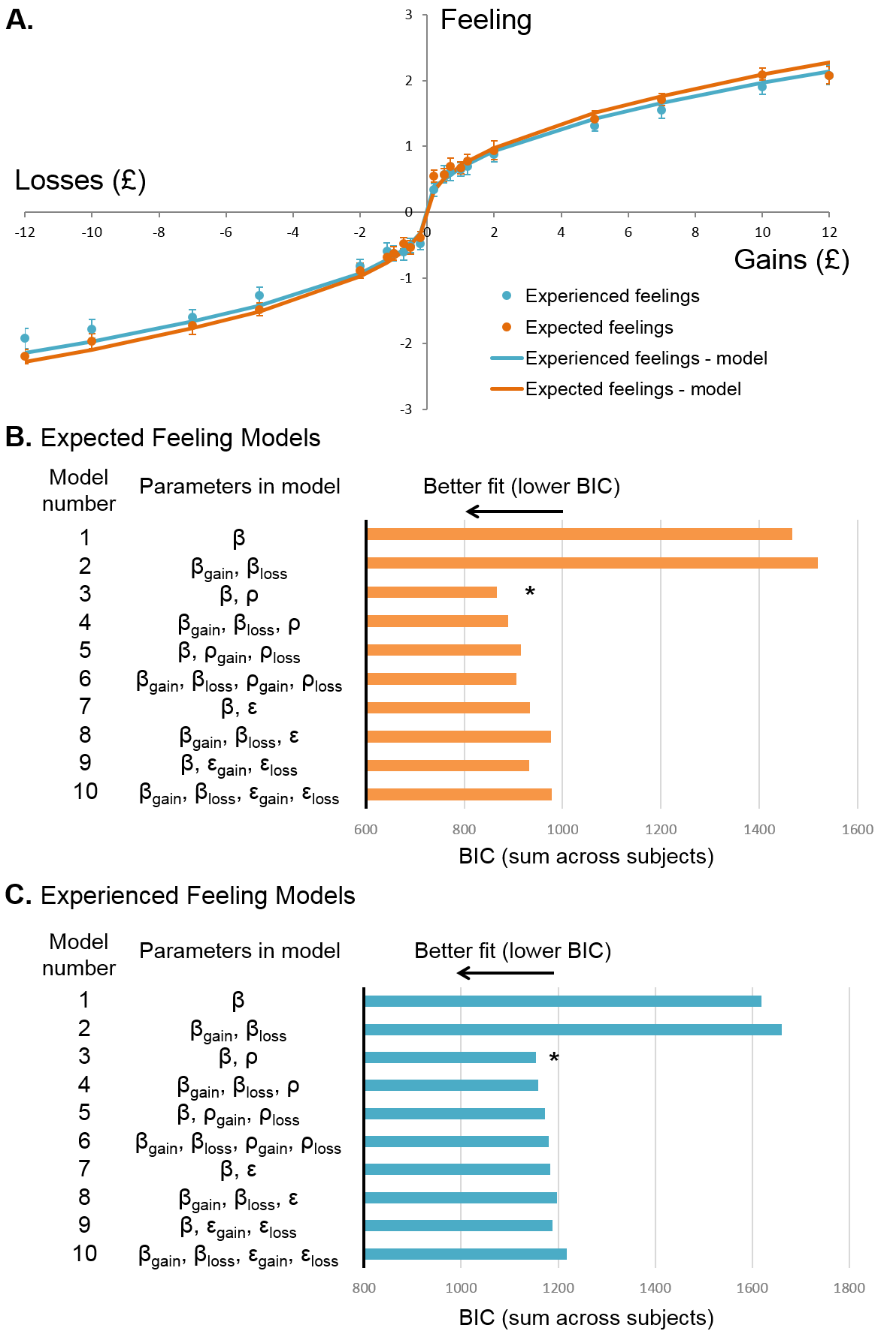

Fig. S4. Replication and extension study 1 – "Feeling function" and Feeling Model fits. Feelings data collected on the replication and extension study were fit using the same procedure as the main study. (**A**) Expected and Experienced feelings ratings are plotted for each outcome value as the average rating across participant. Error bars represent SEM. The line representing best fitting Feeling Model 3 is also plotted. Average beta (β) across participants was 0.702 ± SD 0.24 for expected feelings and 0.669 ± SD 0.25 for experienced feelings. Average rho (ρ) was 0.474 ± SD 0.17 for expected feelings and 0.469 ± SD 0.23 for experienced feelings (both significantly smaller than 1, consistent with diminishing sensitivity of feelings to increasing outcome values: t(19)>10, P<0.001). BIC values, summed across all subjects, for each of ten Feeling Models are plotted separately for (**B**) Expected feelings ratings and (**C**) Experienced feelings ratings. This replicates the finding of

DOI: 10.1177/0956797616634654

the main study (main text **Fig. 2** and **Fig. 3**) that Feeling Model 3 was the most parsimonious model and extends it to a situation in which the impact of gains and losses on feelings is evaluated during the same trial.

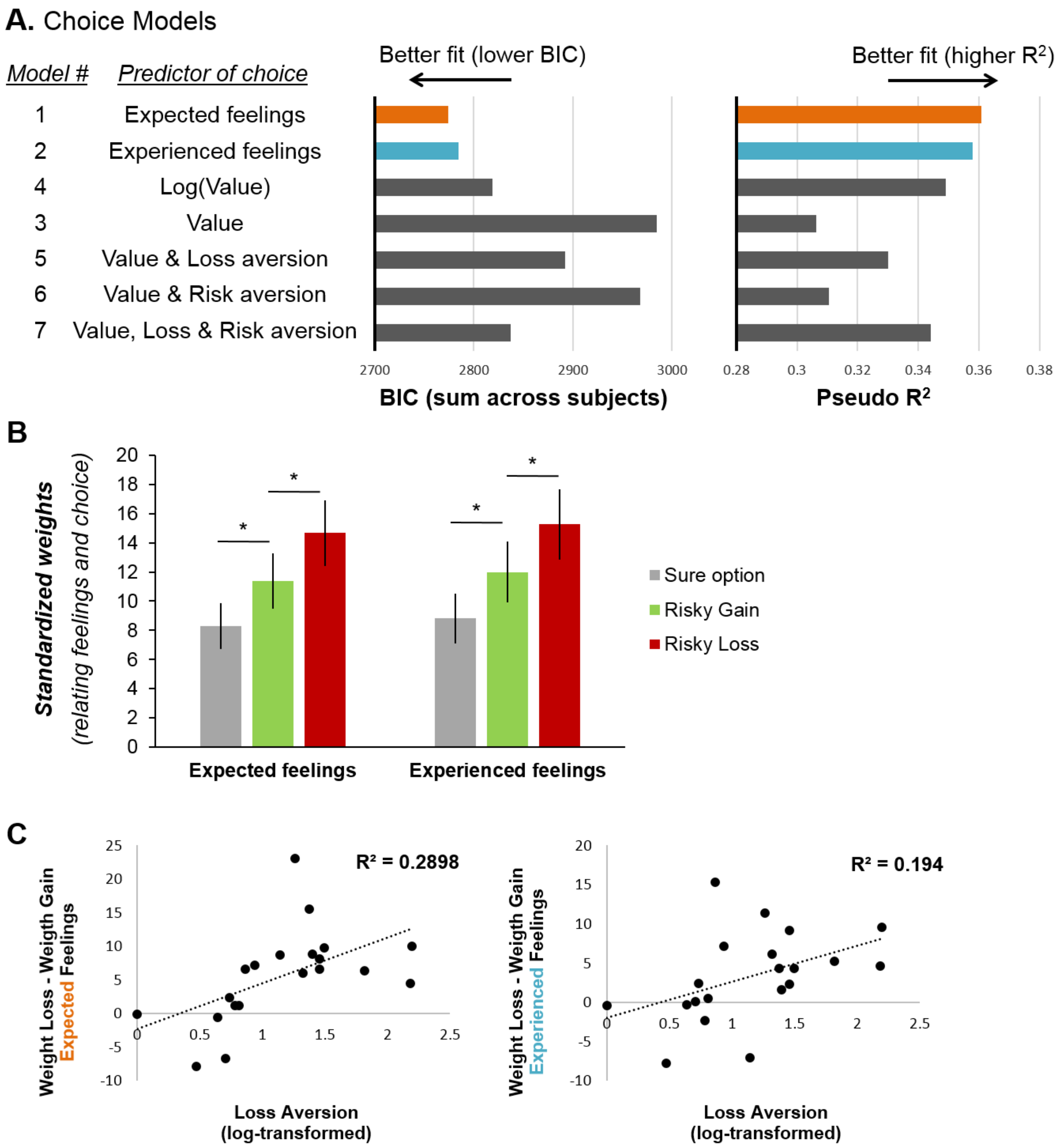

Fig. S5. Replication and extension study 1 – Choice Models. Using the same procedure as in the main study (main text **Fig. 4**), choices on the gambling task were entered in logistic regression models with expected feelings, experienced feelings, or various value-based regressors as predictors. Replicating our findings, BIC scores indicated that derived feelings predicted choice better than all other value-based models (**A**), with feelings about losses weighted more during a decision than feelings about gains (**B**). When running an additional Choice Model where both raw feelings and values were added as predictor of choice (similar to main text **Fig. 5**), thereby allowing us to examine the predictive weight of feelings on choice while controlling for value, we replicated our finding that the extent to which participants overweigh their feelings about losses relative to gains during choice predict individual differences in loss aversion (**C**). Two-tailed paired t-tests: * P<0.05.

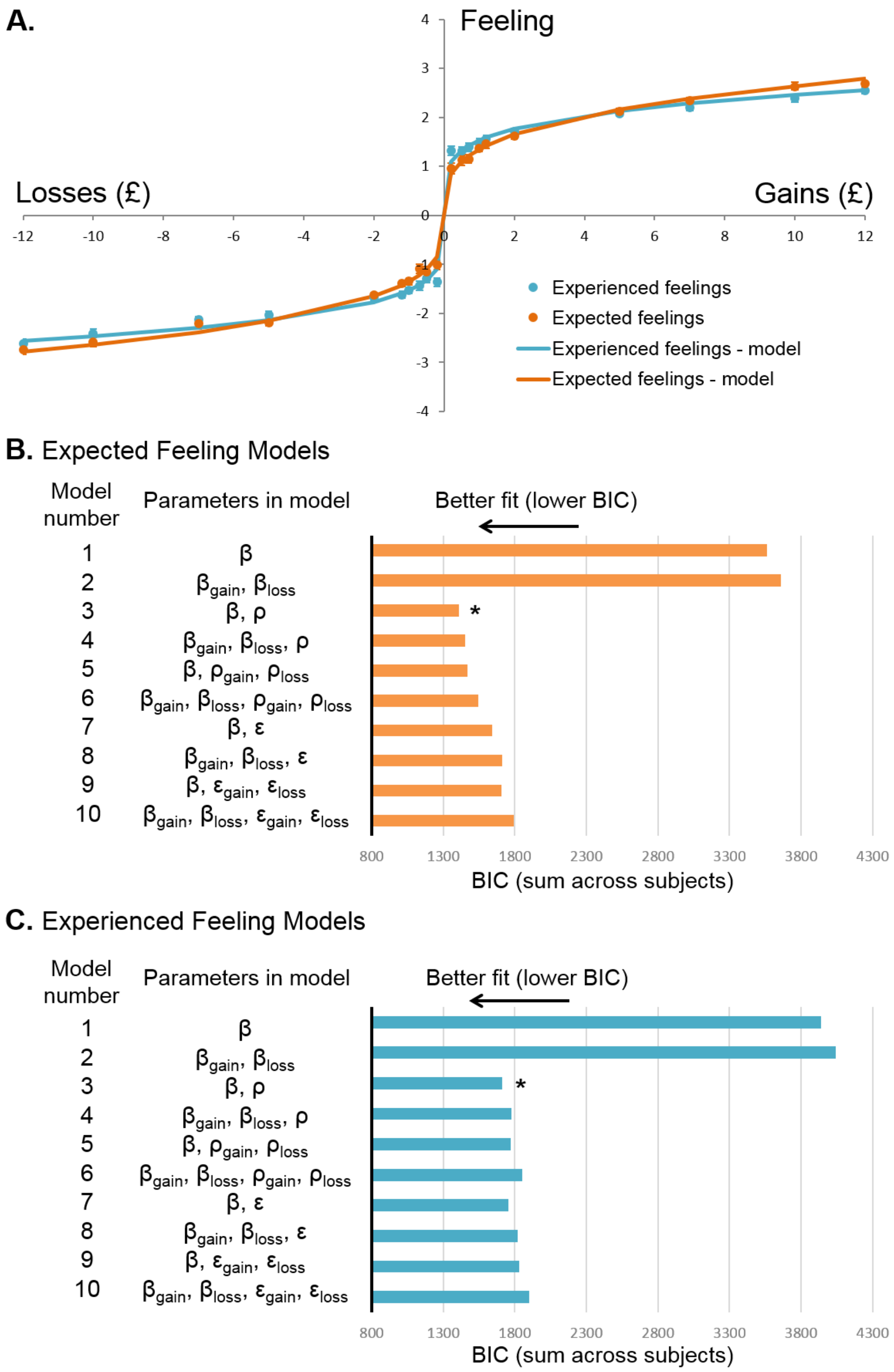

Fig. S6. Replication and extension study 2 – "Feeling function" and Feeling Model fits. Feelings data collected on the second replication and extension study were fit using the same procedure as the main study. The only difference from the main study was the use of a unipolar rating scale to measure reported feelings. (**A**) Expected and Experienced feelings ratings are plotted for each outcome value. Error bars represent SEM. The line representing best fitting Feeling Model 3 is also plotted. Average beta (β) across participants was 1.339 ± SD 0.36 for expected feelings and 1.509 ± SD 0.34 for experienced feelings. Average rho (ρ) was 0.299 ± SD 0.18 for expected feelings and 0.215 ± SD 0.16 for experienced feelings

DOI: 10.1177/0956797616634654

(both significantly smaller than 1, consistent with diminishing sensitivity of feelings to increasing outcome values: t(29)>20, P<0.001). BIC values, summed across all subjects, for each of ten Feeling Models are plotted separately for (**B**) Expected feelings ratings and (**C**) Experienced feelings ratings. This replicates the finding of the main study (main text **Fig. 2** and **Fig. 3**) that Feeling Model 3 was the most parsimonious model and extends the finding to cases where the impact of losses and gains on feelings is reported on a unipolar rating scale.

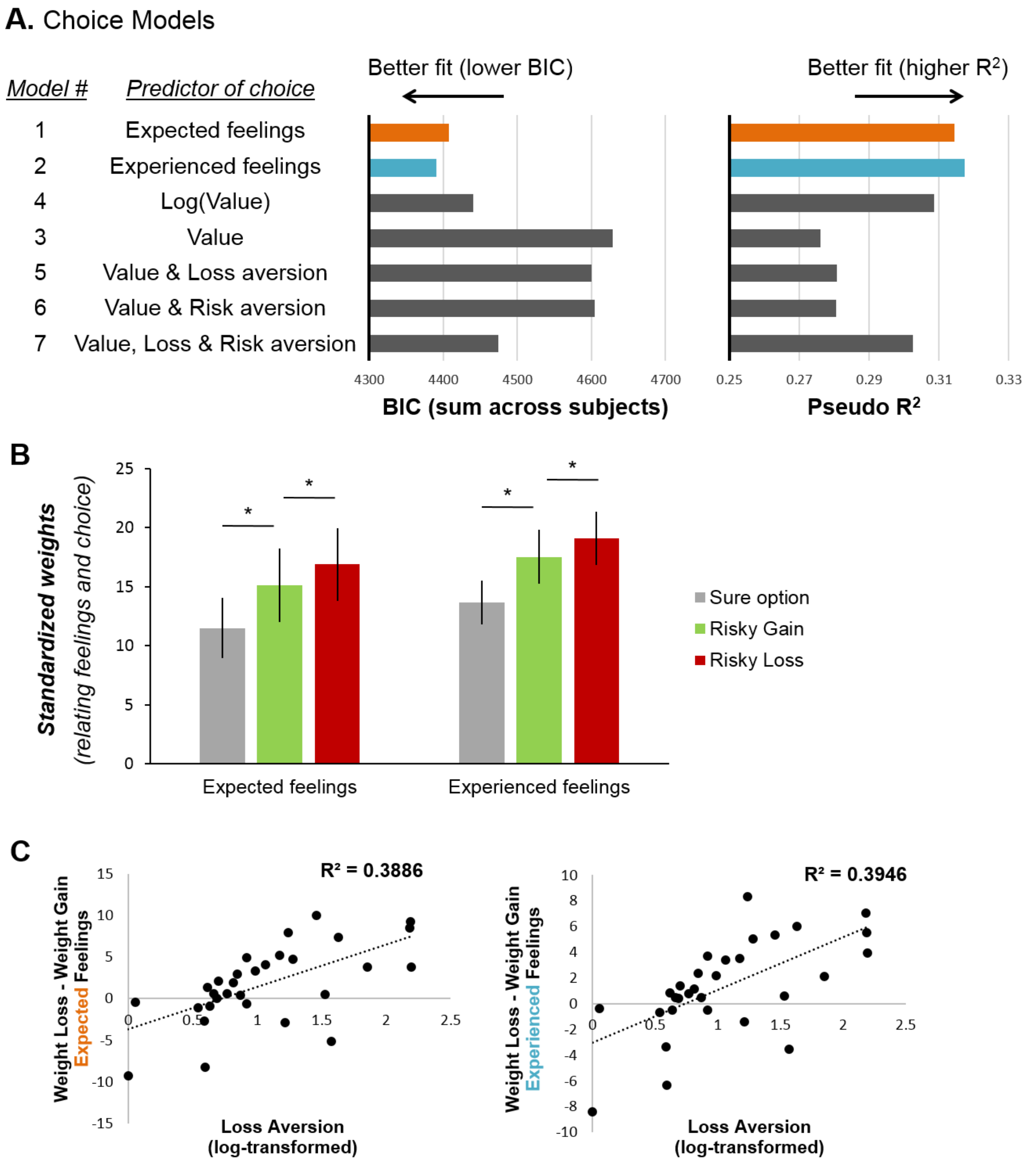

Fig. S7. Replication and extension study 2 – Choice Models. Using the same procedure as in the main study (main text **Fig. 4**), choices on the gambling task were entered in logistic regression models with expected feelings, experienced feelings, or various value-based regressors as predictors. Replicating our findings, BIC scores indicated that derived feelings predicted choice better than all other value-based models (**A**), with feelings about losses weighted more during a decision than feelings about gains (**B**). When running an additional Choice Model where both raw feelings and values were added as predictor of choice (similar

DOI: 10.1177/0956797616634654

to main text **Fig. 5**), thereby allowing us to examine the predictive weight of feelings on choice while controlling for value, we replicated our finding that the extent to which participants overweigh their feelings about losses relative to gains during choice predict individual differences in loss aversion (**C**). Two-tailed paired t-tests: * *P*<0.05.

Table S1. Mean R^2 values associated with each Feeling Model, separately for each method of calculating feelings

		Expected feelings		Experienced feelings	
Model #	Name of parameters	Sum of BICs	Mean R^2	Sum of BICs	Mean R^2
1	β	6625.7	0.720	6561.1	0.637
2	β_{gain}, β_{loss}	6731.5	0.731	6695.0	0.648
3	β, ρ	**5716.1**	0.804	**5594.0**	0.744
4	β_{gain}, β_{loss}, ρ	5792.2	0.814	5628.4	0.758
5	β, ρ_{gain}, ρ_{loss}	5793.4	0.814	5685.6	0.753
6	β_{gain}, β_{loss}, ρ_{gain}, ρ_{loss}	5938.8	0.819	5758.4	0.764
7	β, ε	5833.3	0.800	5674.7	0.742
8	β_{gain}, β_{loss}, ε	5905.1	0.811	5757.2	0.752
9	β, ε_{gain}, ε_{loss}	5947.7	0.808	5723.9	0.755
10	β_{gain}, β_{loss}, ε_{gain}, ε_{loss}	6069.4	0.814	5851.3	0.761

The impact of outcomes on feelings was computed using three different methods: as the change from the rating associated with zero outcome (i.e., the rating associated with not winning or not losing the equivalent amount – zero baseline), as the change from the mid-point of the rating scale, or as the change from the previous rating. All feeling models were then fit to these feelings data. For all feeling models the zero baseline resulted in the best fit. Note, feeling change compared to previous trial feeling could only be computed for experienced feelings, as actual feelings are not measured during expected feelings blocks. Bold indicates the best fitting model.

DOI: 10.1177/0956797616634654

Table S2. Feeling Models

Model #	Number of parameters	Name of parameters	Expected feelings: Sum of BICs	Expected feelings: Mean R^2	Experienced feelings: Sum of BICs	Experienced feelings: Mean R^2
1	1	β	6625.7	0.720	6561.1	0.637
2	2	β_{gain}, β_{loss}	6731.5	0.731	6695.0	0.648
3	2	β, ρ	**5716.1**	0.804	**5594.0**	0.744
4	3	β_{gain}, β_{loss}, ρ	5792.2	0.814	5628.4	0.758
5	3	β, ρ_{gain}, ρ_{loss}	5793.4	0.814	5685.6	0.753
6	4	β_{gain}, β_{loss}, ρ_{gain}, ρ_{loss}	5938.8	0.819	5758.4	0.764
7	2	β, ε	5833.3	0.800	5674.7	0.742
8	3	β_{gain}, β_{loss}, ε	5905.1	0.811	5757.2	0.752
9	3	β, ε_{gain}, ε_{loss}	5947.7	0.808	5723.9	0.755
10	4	β_{gain}, β_{loss}, ε_{gain}, ε_{loss}	6069.4	0.814	5851.3	0.761

Ten different models were fit to the feelings data in order to best explain its relationship to amount lost and gained (see Methods for exact equations). All models were run separately on expected and experienced feelings. Bayesian Information Criterion (BIC) scores were summed across subjects and R^2 values averaged across subjects. Smaller BIC values and higher R^2 values are indicative of better model fit. Note that BIC values cannot be directly compared between expected and experienced feelings models because the numerical values of the dependent variables are different. R^2 alone cannot be used to determine the best fitting model as it does not account for the number of parameters.

Table S3. Comparison between Feeling Model 3 and Feelings Models 4 to 6

	Expected feelings: Number of subjects (/56)	Expected feelings: BIC difference	Experienced feelings: Number of subjects (/56)	Experienced feelings: BIC difference
Model 3 > Model 4	46	-76.1	42	-34.4
Model 3 > Model 5	46	-77.3	44	-92.2

Model 3 > Model 6	50	-222.6	47	-163.1

Feeling Model 3 performed better than Feeling Models 4, 5, and 6 with additional parameters. The table shows the number of subjects for which Model 3 performed better than the compared model, as well as the statistics for the BIC difference between the two models (BIC_{model3} – $BIC_{comparison\ model}$). Negative values indicate that Feeling Model 3 was more parsimonious (had a lower BIC).

Table S4. Predictive value of Choice Models

Model #	Predictor of choice	Pseudo R^2
1	Expected feelings	**0.31**
2	Experienced feelings	**0.31**
3	Log(Value)	**0.30**
4	Value	**0.26**
5	Value & Loss aversion	**0.27**
6	Value & Risk aversion	**0.27**
7	Value, Loss & Risk aversion	**0.28**

Choice Models using feelings derived from each subject’s feeling function predicted choice better than Choice Models using value or value-derived functions. Note that all Choice Models were run on the exact same half of the choice data and that feeling and value functions were extracted from separate, independent data. Therefore, these Choice Models are directly comparable. Given that all models have the same number of parameters (ω_G, ω_L and ω_S, representing the weights associated with gain, loss and sure option on choice, respectively), higher pseudo R^2 value indicate better model fit.

Table S5. Choice Models where losses and gains are weighted differently perform better

	Expected Feelings		Experienced Feelings	
	BIC	R^2	BIC	R^2
Losses and gains weighted differently (ω_S, ω_G, ω_L)	17092	0.30	17156	0.30
Losses and gains weighted together (ω_S, ω_{GL})	19594	0.18	19519	0.18

DOI: 10.1177/0956797616634654

Separate logistic regressions models were run on all choice trials to predict choice from feelings (either expected or experienced). Specifically, to demonstrate that feelings for losses and feelings for gains had a different weight on choice, choice models where losses and gains are weighed differently were compared to choice models where both losses and gains are given the same weight ω_{GL}. This revealed that choices are predicted significantly better when feelings for losses and feelings for gains are assigned different weights.

Table S6. Weight of feelings on choice, while controlling for value, separated by gamble type

		Expected feelings		Experienced feelings	
		Mixed gambles	Gain/Loss-only gambles	Mixed gambles	Gain/Loss-only gambles
Weight of feelings about **gains** on choice, controlling for value (±SD)		-0.202 (±6.01)	1.976 (±5.92)	0.388 (±4.25)	3.045 (±13.51)
Weight of feelings about **losses** on choice, controlling for value (±SD)		4.017 (±8.11)	2.246 (±4.43)	2.671 (±5.14)	0.029 (±6.34)
T-test Loss vs Gain	t(53)=	2.843	0.302	2.709	1.522
	P=	0.006	0.763	0.009	0.134

Both raw feelings (i.e. reported feelings relative to baseline rather than those derived from the feeling function) and objective values were added as predictor of gambling choice in the same logistic regression, separately for each gamble type. The weights of feelings about gains and losses were extracted from each regression, averaged across subjects, and compared using a paired t-test. Data from two participants were excluded because the logistic regression models could not be fit and resulted in aberrant parameter values.
